# Simple Sequence Repeats (SSRs) and Telomeric Analysis in Somatic Organs of Reproductive and Non-Reproductive Castes of Termite *Reticulitermes chinensis*

**DOI:** 10.3390/biology14020166

**Published:** 2025-02-06

**Authors:** Haroon, Zahid Khan, Wasim Javaid, Lian-Xi Xing

**Affiliations:** 1 College of Forestry and Landscape Architecture, South China Agricultural University, Guangzhou 510642, China; haroonsbbu@gmail.com (H.); wasimjavedwasi@gmail.com (W.J.); 2 College of Life Sciences, Northwest University, Xi’an 710069, China

**Keywords:** *Reticulitermes chinensis*, FISH, SSRs, telomere, aging

## Abstract

The study of *Reticulitermes chinensis* offers valuable insights into insect aging and longevity, focusing on telomere biology and simple sequence repeats (SSRs). Telomeres, the protective cap at chromosome ends, are often linked to cellular aging and lifespan. Through transcriptomic analysis using the RepeatExplorer tool, a total of 10,740 SSR loci were identified, encompassing di-, tri-, tetra-, penta-, and hexa-nucleotide motifs. Among these, tri-nucleotide repeats were the most prevalent (2702), with prominent motifs including AC/GT (21.91%), AAG/CTT (8.49%), and AGC/CTG (8.2%). The identified SSRs serve as valuable genetic markers for taxonomy, phylogenetic, and population genetics. A telomeric sequence array featuring the TTAGG repeat motif was also discovered, with fluorescence in situ hybridization (FISH) confirming its localization at chromosome ends. Telomere lengths *R. chinensis* ranged from tens to hundreds of kilobases but showed no significant correlation with lifespan differences among termite castes. All castes had the same telomere length. This finding suggests that *R. chinensis* may possess a unique telomere maintenance mechanism, decoupling telomere length from aging and challenging the conventional view that shorter telomeres are indicative aging. It is hypothesized that telomerase activity plays a critical role in preserving telomere integrity in this species. These findings underscore the complexity and evolutionary adaptations of telomere biology in social insects. Moreover, the variation and organization of SSRs in *R. chinensis* provide a rich genetic resource for genome mapping, evolutionary research, and population genetics. This study sheds light on telomere dynamics and genetic diversity in termites, opening new pathways for research in evolutionary biology and the molecular mechanisms of aging.

## Simple Summary

This study investigates the transcriptome of *Reticulitermes chinensis*, focusing on the roles of simple sequence repeats (SSRs) and telomeres in the aging process. Using the Illumina HiSeq 4000 platform, 103 million sequence reads were generated and processed, yielding 184,436 unigenes. Genomic repeat sequences were assembled and analyzed using tools such as Trinity and RepeatExplorer. The findings underscore the critical role of telomere integrity in cellular aging and shed light on the molecular mechanisms underlying longevity in social insects.

## 1. Introduction

Simple sequence repeats (SSRs) or microsatellites are influential tools for determining genetic divergence between population structures and estimating genetic diversity across taxa [1,2,3] that are effective for conservation strategies [4]. SSRs are genomic elements characterized by the tandem repetition of short nucleotide motifs (one to six base pairs in length) [5]. Despite constituting approximately 1% of the genomic content in most sequenced organisms, SSRs have traditionally been classified as nonfunctional DNA owing to their perceived lack of coding potential [6,7]. Furthermore, SSRs exhibit remarkable polymorphism, demonstrating substantial variation both within and among populations. This characteristic renders them invaluable for diverse scientific applications. In taxonomic studies, SSRs serve as powerful tools for distinguishing closely related species. Their utility extends to phylogenetic analyses, where they elucidate evolutionary relationships [1,2,3]. Furthermore, in population genetics, SSRs play a crucial role in evaluating genetic variation and population structure [1,2,3]. The exceptional variability of SSRs enables researchers to delineate lineage and migration patterns with precision, offering significant insights in ecological and evolutionary investigations.

However, recent genomic analyses have revealed a considerable presence of SSRs in various species [8]. Notably, the genome of the body louse *Pediculus humanus* exhibits an SSR composition of 10.52% [9]. In comparison, the Californian leech *Helobdella robusta* demonstrates 6.36% SSR, and the penaeid shrimp *Litopenaeus vannamei* has the most substantial SSR representation observed in animal genomes, with an SSR composition of approximately 23.93% [10].

Additionally, preliminary genomic sequences of other penaeid shrimp species indicate a significant SSR presence, estimated at approximately 10% [10,11]. Because of its high polymorphism, SSR is a useful tool for taxonomic, phylogenetic, and population genetic studies [5,12]. These observations prompt a reevaluation of SSR functions, particularly concerning their contribution to the adaptive processes and evolutionary trajectories of species with high SSR densities. The mechanisms underlying the origin and proliferation of SSRs within these genomes remain an area of active research.

Over the past several decades, the scientific community has been rigorously investigating suitable model organisms to elucidate the function of telomerase in the aging process [13,14,15]. Telomere length maintenance is primarily facilitated by telomerase, a ribonucleoprotein reverse transcriptase enzyme predominantly expressed in stem cells, germ cells, and regenerating tissues. However, somatic cells exhibit insufficient telomerase activity to sustain telomere length indefinitely, with most tissues demonstrating minimal telomerase levels [13,14,15]. As a result, telomeres in most somatic tissues undergo progressive shortening with age, effectively functioning as a biological chronometer that records the passage of time through successive cell divisions. The process of telomere shortening is further influenced by various factors, including recombination, epigenetic regulation, genetic determinants, and oxidative stress [13]. The capacity of telomerase to counteract these influences is limited, contributing to the overall decline in telomere length over time. Telomeres, the intricate DNA–protein complexes situated at the termini of chromosomes, are composed of repetitive TTAGGG sequences that span from a few to approximately 15 kilobases. These structures are crucial in maintaining genomic stability and cellular longevity [13]. Extensive research has conclusively demonstrated that telomeres undergo progressive shortening with each successive cell division, a phenomenon attributed to the end-replication problem inherent in linear chromosomes. The functionality of telomeres is intricately dependent on two key factors: the presence of a minimal threshold length of TTAGGG repeats and the association of specific telomere-binding proteins. These proteins form a protective cap, shielding the chromosome ends from degradation and preventing them from being recognized as DNA double-strand breaks [13]. The interplay between telomere length dynamics and the associated protein complexes is fundamental to cellular senescence, organismal aging, and the development of various age-related pathologies, including cancer [15]. Furthermore, studies have explored the connection between telomere length and adverse life circumstances, including stress and unhealthy lifestyle behaviors and its implications for longevity [15]. In rodents, notably mice, the observation of significantly elongated telomeres correlates with the upregulation of telomerase expression, suggesting that telomere shortening may not be a limiting factor in their natural lifespan [13,14,16,17]. Despite this, the field of insect telomere biology remains comparatively under-investigated, especially concerning the roles of telomeres and telomerase in aging and longevity [18,19]. In the ant species *Lasius niger*, sexual dimorphism in telomere length is evident, with female queens and workers exhibiting substantially longer telomeres than their male counterparts. Intriguingly, no significant telomeric variation is discernible between the queen and worker castes [20,21].

In contrast, the honeybee *Apis mellifera* shows consistent telomerase activation within the somatic cells of queens from the developmental stages to maturity. This activation contrasts with the negligible telomerase activity observed in adult male drones and workers [21,22,23]. These findings have spurred further investigation into the potential link between telomerase activity and increased longevity in eusocial insects, particularly termites [24]. Compared with castes, termite alates exhibit a significantly higher abundance of telomerase and enzymatic activity in their somatic organs [25]. Exploring telomere dynamics in termites offers a unique perspective on aging mechanisms, potentially divergent from well-characterized pathways in vertebrates. As research progresses, it may provide novel insights into the molecular underpinnings of longevity and its evolutionary implications in the insect kingdom.

In the intricate social structure of termite colonies, the differential expression of genes plays a pivotal role in the development and longevity of various castes of termites and other insect species, such as *Drosophila* [26] and grasshopper *Romalea microptera*, with an increase in mortality and a decrease in breeding mechanisms during aging [27], whereas fruit flies [27] and Cowpea weevil *Callosobruchus maculatus* experience survival costs during mating [28]. Insulin and juvenile hormones affect the yolk precursor vitellogenin in the honeybee *A. mellifera* [29,30,31], influencing immunity, oxidative stress, and lifespan, potentially contributing to the longevity of queens. Similarly, termite alates exhibit a long lifespan coupled with lifelong fecundity relative to castes [25,32,33]. The cellular environment uses in situ hybridization to elucidate gene expression patterns, employing labeled RNA or DNA probes that are detectable using antibodies [34,35,36]. This dual detection system reveals gene expression and the precise localization of mRNA within cells [34,35,36]. In the current study, we focused on evaluating the entire caste system of a termite colony, from the primary king and queen to secondary reproductive workers, neotenics, soldiers, and workers [35]. We employed complementary methodologies to dissect the activation mechanisms of telomerase and the resulting telomere lengths. Our focus extends to all developmental stages and castes, encompassing the reproductive cells of both sexes across various ages and organs. To ensure the robustness of our findings, we replicated key experiments within the castes of termite *R. chinensis,* a phylogenetically distant termite species.

This replication allowed us to draw parallels between developmental patterns and longevity of reproductive and worker castes. We investigated the activation dynamics of the telomerase mechanism and telomere length using viva fluorescence in situ hybridization (FISH). We meticulously recorded the abundance and distribution of SSRs across all the developmental stages and castes. This comprehensive approach enabled us to explore the evolutionary consequences and analyze the diversity of termite populations. Our findings contribute to a deeper understanding of the molecular mechanisms that govern caste differentiation and longevity in termites and offer insights into the broader implications of aging pathways in social insects.

## 2. Materials and Methods

### 2.1. Termite Collection and Rearing

Colonies of the termite *Reticulitermes chinensis* were excavated and isolated from their natural habitat in Chengdu between April and May 2014. These colonies were subsequently reared under controlled laboratory conditions, simulating generosity and density, at ambient temperatures ranging from 25 °C to 28 °C (±1 °C) at the Northwest University, Xian, Shaanxi, China. The primary colonies were established in specialized plastic boxes measuring 80 × 65 × 40 mm, filled with pine sawdust, and maintained at 50–60% humidity [37]. Inaugural colonies were constructed using mono-hybrid mating strategies (**♂ × ♀**) to ensure genetic consistency. The selection criteria for experimental use included early stage colonies that exhibited both active and mature characteristics. From these, we selected castes encompassing the primary reproductive king (PK) and queen (PQ), secondary worker reproductive king (SWRK) and queen (SWRQ), and non-reproductive male (WM) and female (WF) worker castes. Our comparative analysis mainly focused on telomerase activity (TA) and telomere length (TL) across the aforementioned castes derived from mature laboratory colonies [21]. This approach has facilitated a comprehensive examination of the telomeric landscape, providing insights into the molecular mechanisms underlying aging and longevity within termite societies.

### 2.2. Experimental Samples

Termite *Reticulitermes chinensis* castes were subjected to meticulous micro-dissection after six years of rearing. The dissection process was carefully timed to immediately follow the six-year mark to ensure the consistency and relevance of the data. Tissue samples comprising the heads, thoraxes, and legs were extracted from PK, PQ, SWRK, SWRQ, WM, and WF castes. These tissues were then amalgamated to form composite samples for each caste that was designated for FISH analysis. This technique allows the visualization of specific genetic sequences within the context of intact tissue, providing a spatial understanding of gene expression [21]. Head tissues from the castes were pooled for total RNA extraction. This step is critical for subsequent transcriptomic analyses to elucidate gene expression profiles pertinent to each caste. We adopted a rigorous replication strategy to ensure the reliability and reproducibility of our findings. Each caste sample underwent three technical replicates to minimize any potential errors or variations inherent to the experimental procedures. Additionally, five biological replicates were pooled, further strengthening the statistical power of this study and allowing for the robust interpretation of the telomeric and transcriptomic data (Table S1).

### 2.3. Total RNA Extraction, cDNA Synthesis, and Illumina Sequencing

Tissue samples from *R. chinensis* were meticulously dissected and promptly cryopreserved in liquid nitrogen to stop enzymatic activity. RNA extraction was performed using TRIzol reagent, followed by quality assessment using an Agilent 2100 bioanalyzer to procure high-quality RNA suitable for next-generation sequencing (NGS) on the Illumina platform (Agilent Technologies, Palo Alto, CA, USA). The homogenization process employed both the Lysate RZ component of the TRIzol reagent and the RNA-simple Total RNA Kit to ensure thorough cell lysis while protecting the RNA from enzymatic degradation (Tiangen Biotech “Beijing” Co., Ltd., Beijing, China) [37]. Homogenized tissue samples were maintained at a controlled temperature range of 15–30 °C to facilitate the complete dissociation of nucleoprotein complexes. Subsequent steps included the transfer of homogenates to RNase-free centrifuge tubes, phase separation, RNA precipitation, and the removal of proteins and other contaminants. The extracted RNA was then quantified, and its purity was confirmed by assessing protein contamination at an absorbance ratio of 260/280 nm and salt contamination at 260/230 nm using a NanoReady spectrophotometer (NanoReady “Model: F-1100” made in China) and validated through gel electrophoresis. Integrity-verified RNA was conserved at −80 °C for future analytical applications [38].

Complementary DNA (cDNA) was synthesized using the NEB-Next Prep-Kit for Illumina (NEB) kit for Illumina (NEB) sequences. After reverse transcription, the cDNA fragments were subjected to size selection and purification using a QiaQuick PCR purification kit. The poly (A) ends of the cDNA were adenylated and ligated to the Illumina sequencing adapters. High-throughput sequencing was performed on the Illumina HiSeq 4000 platform, yielding high-quality reads with an average length of 1.2 million bases, using Gene Denovo Biotechnology Co. Ltd. (Guangzhou, China) to assess the distribution of the ligation product size. For de novo transcriptome assembly, Trinity software suite (trinityrnaseq r2012-04-27) was employed to generate a comprehensive set of unigenes [38]. To analyze genomic repeat sequences, RepeatExplorer, a graph-based clustering tool, was used on the Galaxy web-based platform (http://repeatexplorer.umbr.cas.cz/, accessed on 13 February 2023). This analysis allowed for the quantification of repeat sequence abundance in the genome based on the read count per cluster [39,40].

### 2.4. Simple Sequence Repeat (SSR) Analysis

Sequence reads were annotated using the Illumina HiSeqTM 4000 platform and Trinity software (trinityrnaseq r2012-04-27), encompassing a diverse range of base pair (bp) lengths derived from transcriptome data [38]. GC content analysis and overall quality assessment of the assembled unigenes were conducted using the MISA tool (http://pgrc.ipk-gatersleben.de/misa/, accessed on 16 February 2023) to identify SSR in unigenes [41]. The analysis predicted and identified SSRs in the unigenes that had motifs of di-, tri-, tetra-, penta-, and hexa-nucleotides. The amplification products were characterized by consecutively repeating units six or more times (≥6 times) (See Supplementary Materials).

### 2.5. Telomere Detection by FISH

Chromosomal FISH preparations were carefully obtained from termite *R. chinensis* tissue samples. The tissues were initially fixed in a solution with a 3:1 ratio of ethanol to glacial acetic acid, ensuring preservation of the cellular structures, and subsequently stored at −20 °C for stabilization [42]. Chromosome squashes were crafted in a medium containing 50% acetic acid, post-frozen in liquid nitrogen, and the coverslips were detached. Slides were air-dried and stored at 4 °C for long-term preservation [43,44]. For FISH assays, telomeric TTAGG probes were synthesized with the sequences for the forward primer TAGGTTAGGTTAGGTTAGGT and the reverse primer CTAACCTAACCTAACCTAAC. These probes are specific to the telomeric TTAGG repeat sequence, a conserved feature in many ancestral species, and FISH assays were conducted according to the procedures previously described [44,45]. Application of TTAGG_n_ probes resulted in significant enrichment, indicating successful hybridization to the target telomeric regions in the chromosomes of the termite *R. chinensis*. This enrichment was visualized and the probes’ efficacy in binding to telomeric sequences was demonstrated.

Telomeric probes were synthesized using the primers (TTAGG)6 and (TAACC)6 following established protocols [21,44,46]. Polymerase chain reaction (PCR) was conducted without a template, utilizing 100 pmol of each primer and 2.5 units of Taq polymerase in a 100 µL reaction volume [21,44]. The PCR cycling conditions were 30 cycles of denaturation at 95 °C for 60 s, annealing at 50 °C for 1 min, and extension at 72 °C for 3 min, with a final elongation at 72 °C for 10 min. PCR products ranging between 200 bp and 1 kb were purified and labeled with biotin-16-dUTP using the Nick Translation Kit (Roche) according to the manufacturer’s guidelines [21,44]. The labeled probes were precipitated and resuspended in 50% formamide [47].

Chromosomal slides prepared from tissues were treated with RNase A, pepsin, and formaldehyde, followed by dehydration using a graded ethanol series (70%, 90%, and 100%) [48,49]. For hybridization, 25 µL of the DNA-labeled solution was applied to each slide, which was then denatured at 80 °C for 3 min and immediately frozen on ice [44,49,50]. The hybridization mixture contained 50% formamide 2×SSC (2×SSC), 50 mM sodium phosphate, 0.1 mg/mL DNA, 0.1 mg/mL yeast RNA, and 5 ng/mL of the labeled telomere probe [44,50]. Slides were incubated overnight in a humidified chamber at 37 °C [44]. After hybridization, the slides were washed in 50% formamide at 37 °C and 2×SSC with 0.05% Tween-20 at pH 7.5 [51,52]. The avidin-FICT/anti-avidin–biotin system was used for immunological fluorescence detection with four rounds of amplification. Vectashield mounting medium (vector) was used, and chromosomes were counterstained with DAPI in an antifade solution to facilitate visualization under a fluorescence microscope [44]. These techniques enable accurate identification of telomeric sequences on chromosomes and offer valuable information on the structure and function of chromosomes. FISH using telomeric probes is a highly effective cytogenetic technique for investigating telomere length and integrity. These factors are important indicators of cellular aging and genomic stability.

## 3. Results

### 3.1. Simple Sequence Repeat (SSR)

**Figure 1:**
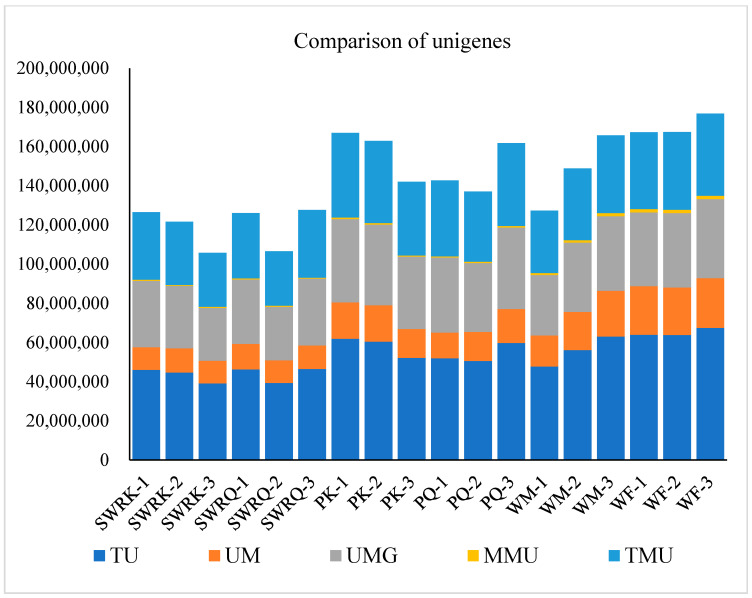
The total number of unigenes (TU), unmapped unigenes (UU), unique mapped unigenes (UMU), multiple mapped unigenes (MMU), and total mapped unigenes (TMU) in different castes of termite *R. chinensis*. PK means primary reproductive king; PQ—primary queen; SWRK—secondary worker reproductive king; SWRQ—secondary worker reproductive queen; WM—non-reproductive male; WF—female worker castes. The *X*-axis represents different castes of the termite species *R. chinensis* (workers and reproductive). The *Y*-axis represents the number of unigenes categorized into various groups based on their mapping status.

**Figure 2:**
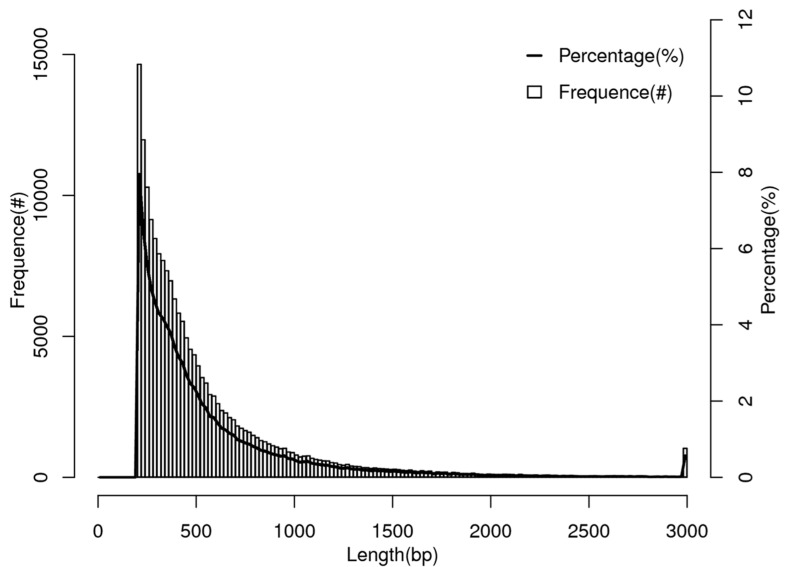
Histogram representing the length distribution unigenes of *R. chinensis*; the total assembled unigenes and length distribution for the identified significant matches. The *X*-axis indicates the sequence length (bp) sizes from >200 nt to >3000 nt. The left side of the *Y*-axis indicates the frequency (♯) and the right side of the *Y*-axis indicates the percentage (%) of unigenes for every given size.

The transcriptomic analysis of *R. chinensis*, yielded a substantial dataset comprising 103,589,264 sequence reads with an average length of 561 base pairs (bp) (Table 1, Figure 1). The high-throughput sequencing was conducted using the Illumina HiSeq 4000 platform, providing a comprehensive view of the termite’s gene expression profile. Furthermore, we identified 184,436 unigenes, representing a non-redundant set of transcript sequences ranging from 201 to 43,214 bp. These unigenes were derived from a 7G transcriptome dataset, highlighting the depth and breadth of the genomic information (Figure 2). The wide range of unigene lengths suggests a diverse array of transcripts, including both short regulatory RNAs and long coding sequences for complex proteins. These extensive transcriptomic data not only provide insights into the genetic makeup of *R. chinensis* but also serve as a valuable resource for comparative genomics, evolutionary studies, and investigations into termite-specific biological processes. The use of advanced sequencing technology and the generation of such a large-scale dataset underscore the power of modern genomic approaches in elucidating the molecular basis of termite biology, with implications for understanding social insect evolution, wood degradation mechanisms, and potential biotechnological applications.

The genomic guanine–cytosine (GC) content of the assembled sequences was determined to be 43.02%, indicative of a high-quality sequence assembly. This metric provides valuable insights into the overall composition and stability of the genome. Furthermore, a comprehensive assessment of genome completeness was conducted using Benchmarking Universal Single-Copy Ortholog (BUSCO) analysis. The results revealed a total of 884 complete BUSCOs, with 860 identified as single-copy (S) genes and 24 as duplicated (D) genes. Additionally, 88 fragmented (F) BUSCOs were detected, and only 6 were missing (M) from the assembly. In total, 978 BUSCO genes were evaluated, providing a robust measure of the genome’s completeness and quality (Figure S1). These findings collectively suggest that the assembled genome sequence is of high quality and exhibits a high degree of completeness, crucial for subsequent genomic analyses and comparative studies.

The unigenes were subjected to BLASTX analysis against multiple databases, including Nr (63,512 unigenes), KEGG (43,609 unigenes), COG (27,664 unigenes), and Swiss-Prot (31,758 unigenes) (Figure S2). The expectation value (e-value) obtained from these analyses serves as a critical metric, particularly in the context of sequence alignment. These results quantify the likelihood of obtaining a match by chance, with lower e-values indicating higher confidence in the alignment’s biological significance. Specifically, a lower e-value suggests that the observed alignment is less likely to have occurred randomly, providing a robust quantitative measure of the alignment’s relevance, to identify biologically meaningful sequence similarities and functional annotations, facilitating a comprehensive understanding of the genetic makeup and potential functional roles of the analyzed unigenes, leveraging diverse databases and employing stringent e-value thresholds, to enhance the accuracy and reliability of gene function predictions, ultimately contributing to a nuanced interpretation of the organism’s genomic landscape.

**Figure 3:**
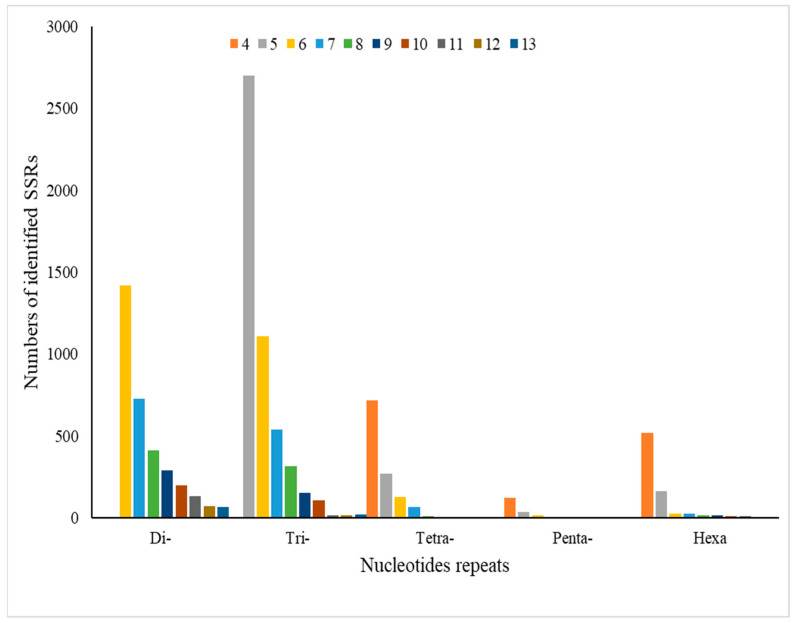
The number of identified SSRs and motif types di-(two), tri-(three), tetra-(four), penta-(five), and hexa-(six) nucleotides repeats. The bar color indicates the repeats of SSR.

**Figure 4:**
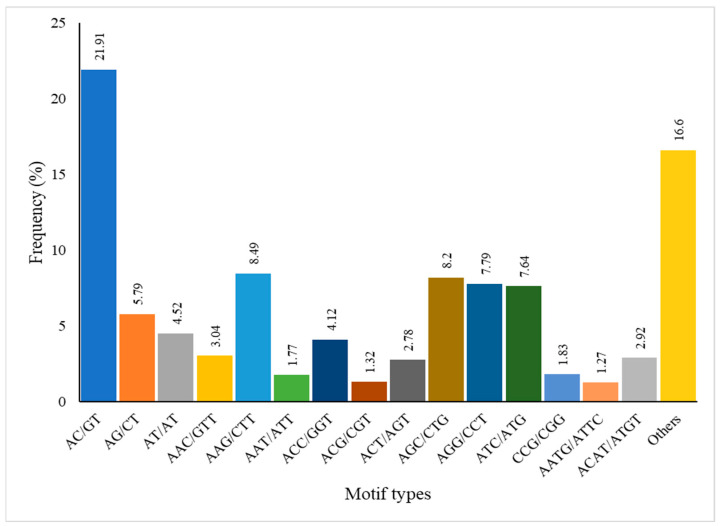
The frequency distribution of SSRs according to the motif and sequence types.

The MISA web tool was used to identify simple sequence repeats (SSRs) within unigenes, yielding 10,740 SSRs with di-(two), tri-(three), tetra-(four), penta-(five), and hexa-(six) nucleotide repeats. The highest number of tri-nucleotide SSRs was found on the fifth chromosome, totaling 2702, followed by 1110 on the sixth chromosome. In contrast, the lowest occurrence of penta-nucleotide SSRs (single instances) was observed on chromosomes 8, 9, and 13 (Figure 3). Further analysis revealed that the AC/GT motif was the most prevalent, constituting (21.91%) of the identified SSRs, followed by unspecified motifs (16.6%) and AAG/CTT and AGC/CTG (8.49%) and (8.2%). The least frequent motifs were AATG/ATTC (1.27%), ACG/CGT (1.32%), and AAT/ATT (1.77%) (Figure 4). These findings provide valuable insights into the genomic architecture of *R. chinensis* and highlight the potential functional significance of SSRs in the genome. The distribution and frequency of SSRs may have implications for genetic diversity, gene expression regulation, and species adaptability.

### 3.2. Identification of Telomerase Structure and Length of Telomeres

**Figure 5:**
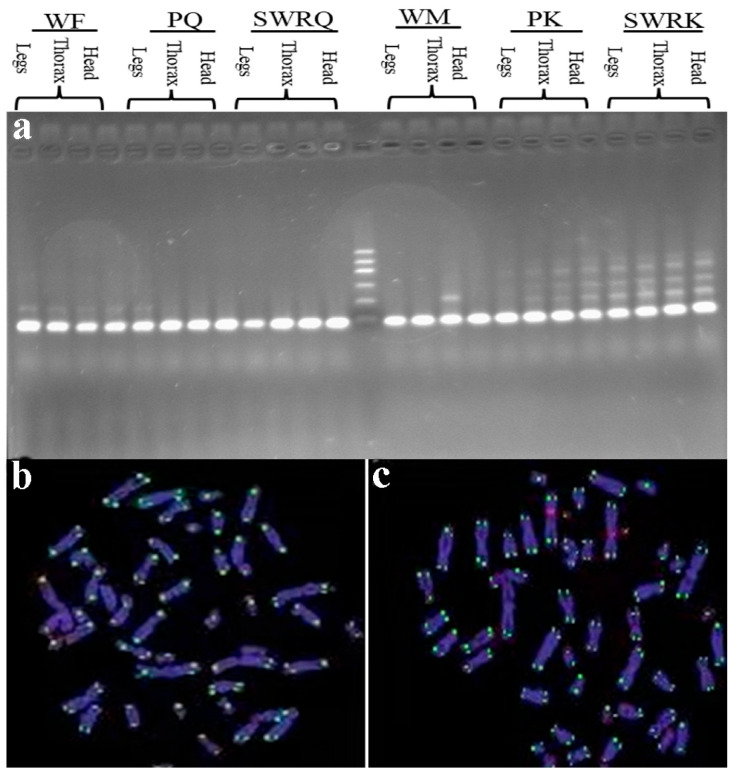
(**a**) Gel analysis and identification of repetitive sequences within the genome. The gel analysis was performed using agarose gel, where genomic DNA was run to identify repetitive sequences within the genome. This experiment allowed us to isolate and confirm the presence of repetitive elements. (**b**,**c**) Fluorescence in situ hybridization (FISH) was conducted using the (TTAGG)_n_ telomeric probe, which generates green signals. The chromosomes were counterstained with DAPI (blue) to highlight both mitotic and meiotic chromosomes in *R. chinensis* castes. (**b**) FISH results indicate the SWRQ (secondary worker reproductive queen), PQ (primary queen), and WF (worker female) castes. (**c**) FISH results illustrate the SWRK (secondary worker reproductive king), PK (primary king), and WM (worker male) castes.

The transcriptomic data of termite *R. chinensis* were subjected to comprehensive analysis using the RepeatExplorer tool, which facilitates the identification of repetitive sequences within the genome. Among the clusters generated by this analysis, one was identified as a telomeric sequence array characterized by the TTAGG repeat motif, a sequence commonly found in telomeres of various insect species. Fluorescence in situ hybridization (FISH) was employed to confirm the presence and chromosomal localization of the telomeric sequence in *R. chinensis*. The FISH analysis revealed a distinctive pattern resembling an oligo-nucleotide ladder, indicative of tandemly repeated telomeric sequences. This characteristic pattern was consistently observed across various castes and tissue types of the species, demonstrating the conserved nature of the telomeric structure within *R. chinensis*. Further validation was achieved by sequencing the elongation products, which confirmed the presence of the (TTAGG)_n_ motif aligned with the expected telomeric sequence. The FISH assay provided clear hybridization signals at the terminal regions of the mitotic chromosomes, corroborating that the telomeres of the termite *R. chinensis* contain the ancestral insect telomeric motif. This discovery enriches our understanding of the chromosomal features of the termite *R. chinensis* and contributes to a broader knowledge of telomere biology in insects, offering potential insights into their evolutionary history and genomic stability mechanisms. The clear visualization of telomeric TTAGG repeats at chromosome ends, as depicted in Figure 5, shows how well the genomic and cytogenetic approaches work together to describe these important genomic elements.

Arthropod telomere has revealed that the penta-nucleotide sequence repeat (TTAGG)_n_ is the predominant and ancestral telomeric motif in the phylum Arthropoda, including insects. This sequence plays a crucial role in maintaining chromosome stability and integrity. In termites, a eusocial insect group within the order Blattodea, the TTAGG telomeric repeat has been confirmed in several species across various families, supporting its ancestral nature within this group. While some insect orders have lost this sequence during evolution, termites have retained it, which may have implications for their genome stability and longevity. Studies have also revealed variations in the number of TTAGG repeats among different termite species and even within individuals of the same species, potentially related to factors such as age, caste, and environmental conditions. Understanding these dynamics could provide valuable insights into termite biology, including aging, reproduction, and social organization while contributing to our broader understanding of arthropod genome evolution and chromosome maintenance mechanisms.

FISH facilitated the precise localization of TTAGG telomeric repeats to the terminal regions of *R. chinensis* chromosomes (Figure 5b,c). This observation was consistent across various castes and tissues within the species, as shown in the figures. The telomeric fragments exhibited considerable lengths, spanning tens to hundreds of kilobases, with the smallest measured fragments exceeding 20 kilobases in length. Notably, this study found no significant variation in telomere length that could be correlated with the varied lifespans of the various castes (Table 2). A further comparison of *R. chinensis* castes at different stages of development, including reproductive (king and queen) and non-reproductive (workers), showed that telomere lengths were quite long, with no clear differences related to reproductive status (Figure 5). These findings suggest that telomere length in the termite *R. chinensis* may not be influenced by caste differentiation or reproductive function, providing unique insight into telomere dynamics within this species. The absence of telomere length disparity among castes, regardless of their developmental potential or longevity, adds complexity to our understanding of the telomere biology in social insects.

## 4. Discussion

The study of polymorphic SSRs is of paramount importance for genetic diversity, mapping, comparative genomics, and marker-assisted selection in breeding programs. SSRs are highly valued because of their variability among individuals within a population, which makes them excellent markers for these applications. Transcriptome sequencing (RNA-Seq), has emerged as a prolific source for discovering SSRs, owing to its ability to generate vast quantities of sequence data encompassing expressed genes and indicative of functional genomic elements. Transcriptome analysis of the termite *R. chinensis* revealed that GC content is an indicator of nucleotide composition and sequence quality (calculated up to 43.02%). This percentage falls within the expected range for a diverse set of organisms, suggesting that the assemblies are of high quality and are suitable for downstream homology-based studies. The identification and characterization of SSRs within the termite *R. chinensis* transcriptome not only facilitate the understanding of the genetic structure and variation within this species but also provide valuable markers for further evolutionary and functional genomic investigations.

The threshold for repeat length and the number of repetitions for a sequence to be considered an SSR can vary [53,54]. Studies often exclude mono-nucleotides, owing to potential sequencing errors, and there is inconsistency in the minimum repeat units considered for di-nucleotides, ranging from three or more [55,56,57]. SSRs are frequently identified from unigenes longer than 1000 base pairs (bp), which may reduce the observed frequency of SSRs because shorter sequences are not considered [58]. The structure and composition of the genome influence the SSR frequency. For instance, the small genome size of the termite genus *Reticulitermes* has been associated with a high frequency of SSRs [32,59]. Different software packages for identifying SSRs can yield varying frequencies of SSR loci owing to differences in algorithms and sensitivity [5]. The abundance of short-iterate repeats plays a role in genomic stability and characteristics such as codon usage, which is essential for proper gene expression. There is a trend where increased repeat length decreases SSR abundance, with longer microsatellites being less common than expected [60]. In the context of *Reticulitermes*, the identified microsatellites are valuable for evolutionary studies, and the development of specific genomic markers that can differentiate between termite castes [61] is crucial for understanding the genetic basis of their social structure and could have implications for pest management strategies. Therefore, the development of specific genomic markers, particularly simple sequence repeats (SSRs), holds significant potential for enhancing pest management strategies by enabling more precise identification and monitoring of termite populations. These molecular tools can be employed to assess genetic diversity, elucidate population structure, and track movement patterns of termites, thereby informing the implementation of targeted and efficient pest control measures. SSR markers offer valuable insights into the genetic makeup of termite colonies, allowing researchers to discern subtle variations between populations and even identify distinct lineages within species. Furthermore, these genomic markers can facilitate the identification of termite species and castes that exhibit heightened resistance to conventional pest management approaches, thus enabling the development of more effective and sustainable control methods. Through leveraging the power of SSR markers, pest management professionals can tailor their interventions to specific termite populations, potentially reducing the reliance on broad-spectrum pesticides and minimizing environmental impact while maximizing the efficacy of control efforts.

Our findings regarding termite telomeres are intriguing. The TTAGG telomeric motif, which is ancestral to insects, appears consistently across various species [62], including termites and their relatives, such as cockroaches, mantises, and stick insects [21]. The observed telomere lengths in termites, ranging from 20 to over 50 kilobases (kb) [20,63], were notably longer than the average insect telomere length, which typically ranges from a few units to 20 kb [20,62,64]. This extended length was even more remarkable, considering that no significant differences were found between castes, tissues, or ages within the termite species studied. The lack of correlation between telomere length and lifespan, especially in the context of eusocial insects such as ants and honeybees, suggests that telomere shortening may not be the primary factor in determining shorter lifespans of worker insects [20,23]. Instead, relatively long telomeres in termites and, by extension, other advanced Polyneoptera might be a pre-adaptation that supports their longer lifespans [64]. This could imply that other biological mechanisms or environmental factors play more critical roles in the eusocial insect aging process and lifespan determination. These long telomeres are maintained across various conditions and do not correlate with the absolute age of workers, challenging the traditional view that telomere shortening is a universal marker for aging in insects.

The evolutionary history of the (TTAGG)_n_ telomere motif in insects is a fascinating subject that intertwines with broader phylogenetic narratives of these organisms. The presence of this motif across various insect orders and even in other arthropods, such as crustaceans, suggests that it is an ancient and conserved element within their genomes [65,66]. The central question is whether the (TTAGG)_n_ motif represents a primordial telomeric sequence present in a common ancestor and is subsequently lost in specific lineages, or whether it emerged independently multiple times throughout insect evolution [67]. The pattern of its distribution, appearing in several branches of the insect phylogenetic tree, supports the former hypothesis that it was an ancestral character that was lost multiple times [68,69]. Telomere repeats typically require telomerase activity. However, the emergence of new simple sequence telomere repeats is considered a rare event owing to the complexity of telomerase function and its evolutionary conservation [70]. This rarity suggests that telomeric sequences are likely to be preserved once established, unless a significant evolutionary pressure or alternative mechanism allows for their alteration or loss. In cases where the simple sequence motif is lost, such as in *Drosophila* [71], the existence of an alternative pathway for telomere elongation, such as transposition of specialized retrotransposons, can compensate for the absence of telomerase [71]. This indicates that the loss of the (TTAGG)_n_ motif is not necessarily detrimental to the organism if other mechanisms can fulfill protective and replicative functions [64]. In *Chironomus*, the evolution of complex telomeres repeats from simpler sequences while retaining some telomeric functions, suggesting a gradual and adaptive transformation rather than a sudden loss [72]. This could represent a form of evolutionary experimentation in which the telomere structure is modified while ensuring the continuation of its essential roles [63,66].

## 5. Conclusions

This research on termite *Reticulitermes chinensis* provides valuable insights into the molecular mechanisms underlying aging and lifespan in social insects, with a focus on telomeres and simple sequence repeats (SSRs). A comprehensive transcriptome analysis revealed an abundance of SSRs, highlighting their potential as effective genetic markers for assessing genetic diversity and evolutionary relationships. Notably, this study identified telomeric sequences with the TTAGG repeat motif and confirmed their chromosomal localization using fluorescence in situ hybridization (FISH). The findings suggest that *R. chinensis* possesses a unique telomere maintenance mechanism, as telomere length does not significantly correlate with lifespan differences among castes. This observation challenges the conventional notion that shorter telomeres are indicative of aging, opening new avenues for investigating telomerase activity and its implications for longevity. This study underscores the complexity of telomere biology in social insects and emphasizes the need for further research into the molecular intricacies of telomere dynamics. This research enhances our understanding of the aging process and contributes significantly to the field of evolutionary biology. The findings have potential applications in various domains, including gerontology, comparative genomics, and the study of social insect biology. Future investigations building upon these results may lead to novel insights into aging mechanisms across different species and potentially inform strategies for promoting healthy aging in humans.

## Figures and Tables

**Table 1:** Total annotated genes with sequenced genes in different castes of *R. chinensis*.

S.No	Samples	Replicates	Sequenced_Total_Genes
1	SWRK*	SWRK-1	140,939
2		SWRK-2	134,023
3		SWRK-3	135,852
4	SWRQ*	SWRQ-1	121,130
5		SWRQ-2	138,925
6		SWRQ-3	123,895
7	PK*	PK-1	140,368
8		PK-2	151,112
9		PK-3	138,891
10	PQ*	PQ-1	133,861
11		PQ-2	151,322
12		PQ-3	147,181
13	WM*	WM-1	169,058
14		WM-2	169,392
15		WM-3	164,677
16	WF*	WF-1	172,488
18		WF-2	173,035
19		WF-3	174,693
20	All		184,413

*PK means primary reproductive king; *PQ—primary queen; *SWRK—secondary worker reproductive king; *SWRQ—secondary worker reproductive queen; *WM—non-reproductive male; *WF—female worker caste. A total of 184,436 genes were identified.

**Table 2:** Karyotypic analyses of the chromosomes of *Reticulitermes chinensis*. All measurements are given in “µm”.

Castes	Head (±SD)	Thorax (±SD)	Legs (±SD)
SWRK	4.08 ± 0.70	2.21 ± 0.17	2.64 ± 0.18
SWRQ	4.09 ± 0.70	2.30 ± 0.23	2.79 ± 0.11
PK	3.71 ± 0.49	2.09 ± 0.06	2.03 ± 0.13
PQ	3.86 ± 0.45	2.14 ± 0.07	2.08 ± 0.16
WM	3.44 ± 0.40	2.00 ± 0.09	1.91 ± 0.12
WF	3.49 ± 0.24	2.03 ± 0.07	1.96 ± 0.14

PK means primary reproductive king; PQ—primary queen; SWRK—secondary worker reproductive king; SWRQ—secondary worker reproductive queen; WM—non-reproductive male; WF—female worker caste.

## Data Availability

This manuscript includes all data generated or analyzed during this study. We uploaded these as Supplementary Files and submitted them to NCBI under the reference PRJNA592596.

## Supplementary Materials

The following supporting information can be downloaded at: https://www.mdpi.com/article/10.3390/biology14020166/s1, Figure S1: BUSCO assessment result of our *R. chinensis* genome; Figure S2: Distribution of e-value in COG, KEGG, Nr and SwissProt; different colour indicates E-values; Table S1: Densitometry readings of *Reticulitermes chinensis* castes with their each gel band.
