# Supplementary material for: Simple Sequence Repeats (SSRs) and Telomeric Analysis in Somatic Organs of Reproductive and Non-Reproductive Castes of Termite Reticulitermes chinensis
Source: Biology (Basel). 2025 Feb 6;14(2):166. doi: 10.3390/biology14020166 (PMC11852100; doi:10.3390/biology14020166)
Supplement: Supplementary file 1 [file biology-14-00166-s001.zip › biology-3334876-supplementary.pdf]

Supplementary Materials

Figures

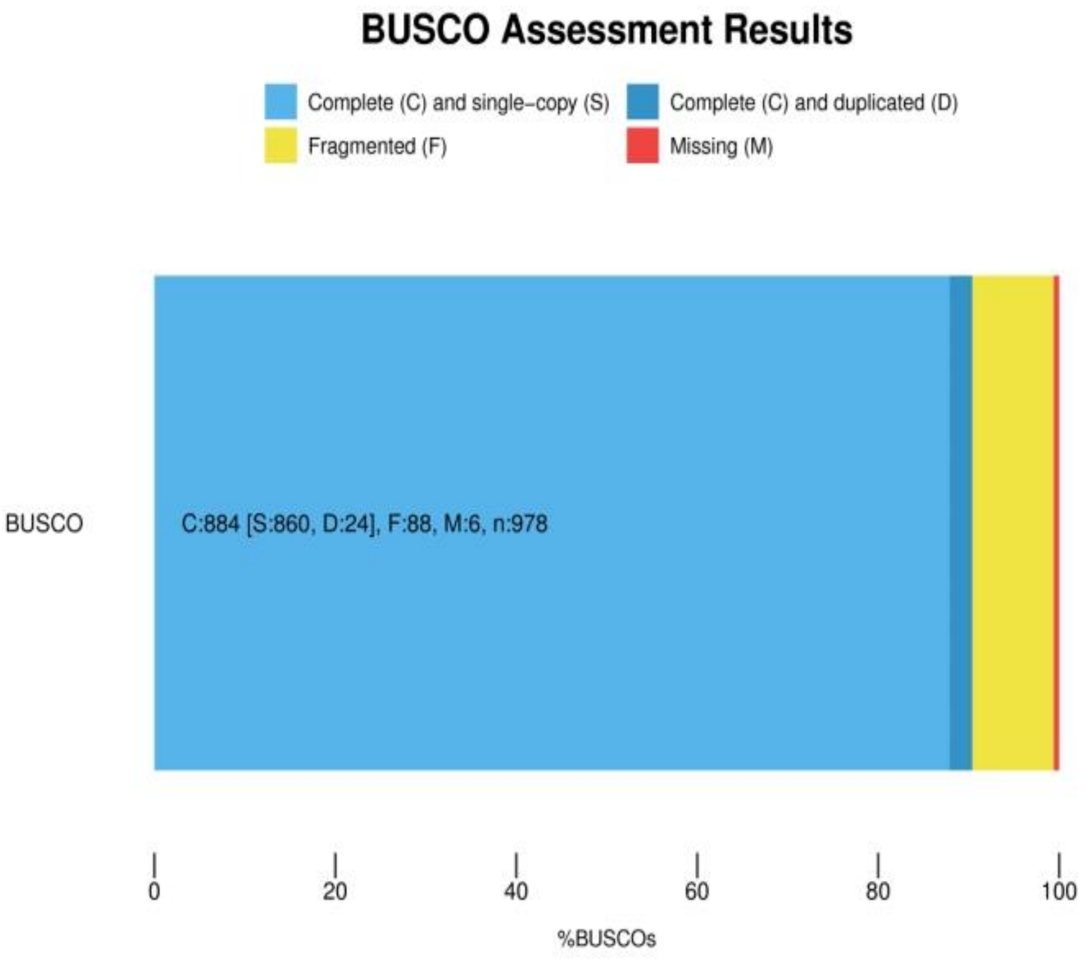

Figure S1. BUSCO assessment result of our *R. chinensis* genome.

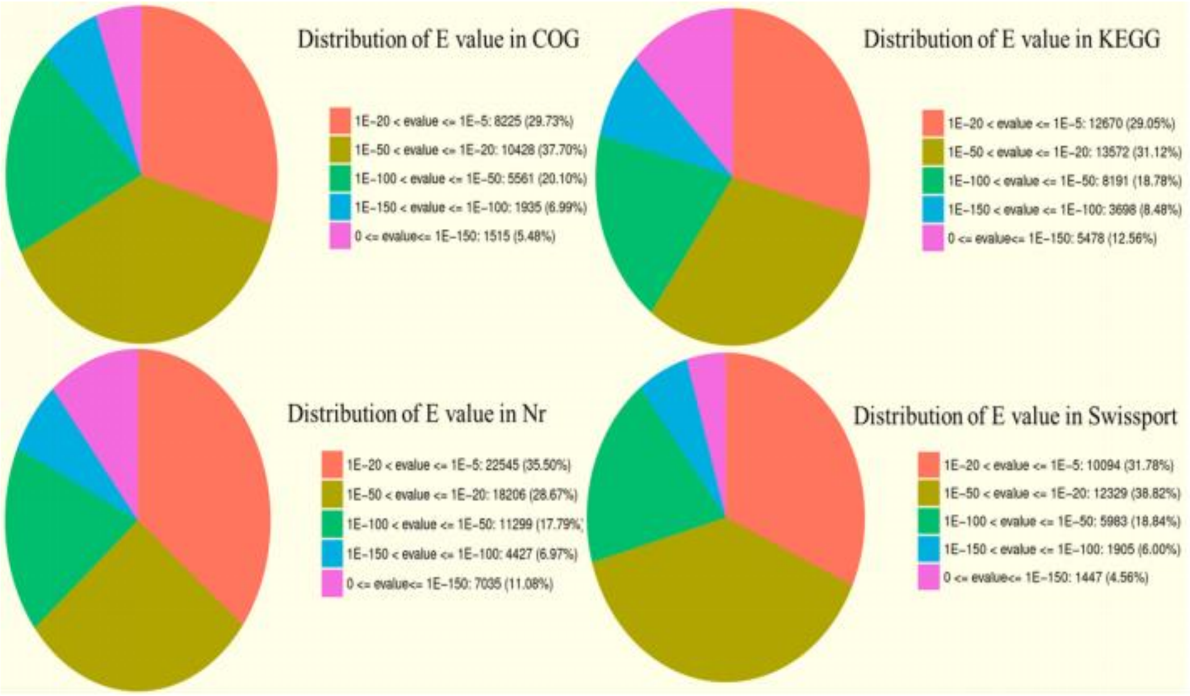

Figure S2. Distribution of e-value in COG, KEGG, Nr and SwissProt; different colour indicates E-values.

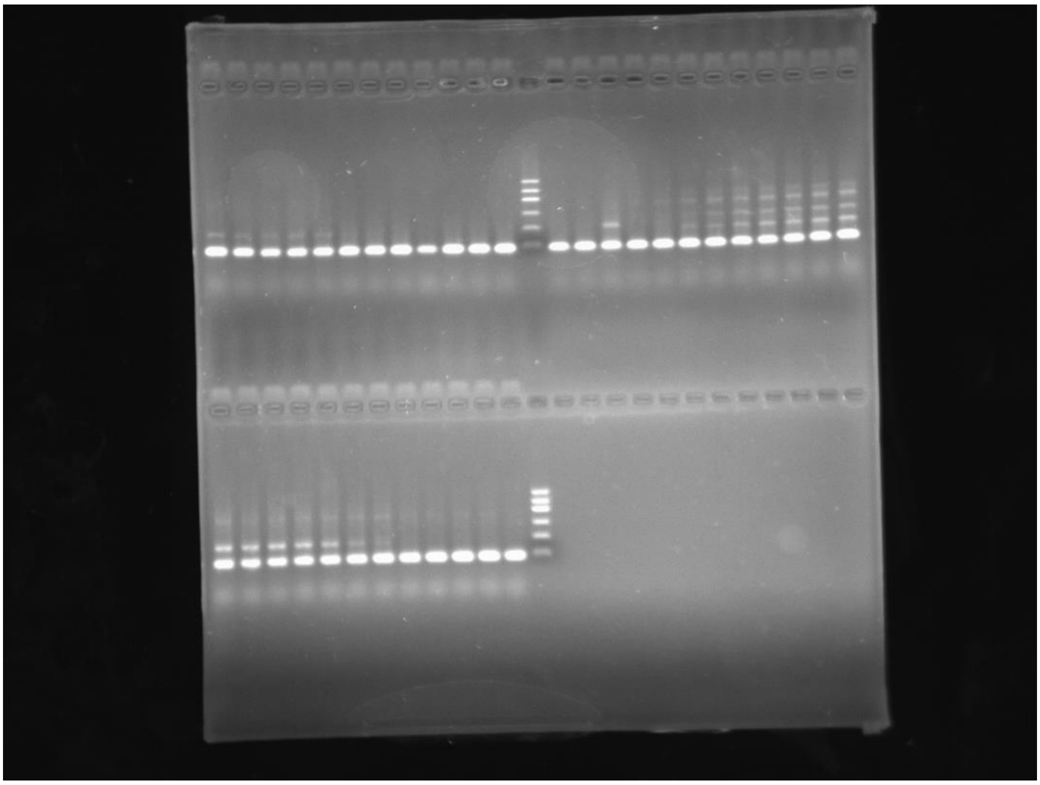

Densitometry readings of *Reticulitermes chinensis* castes with their each gel band.
The details of each band are list in Table S1.

Table S1. Densitometry readings of *Reticulitermes chinensis* castes with their each gel band.

SWRK		SWRK		SWRK	
Head		Thorax		Legs	
Length (mm)	Area (mm^2)	Length (mm)	Area (mm^2)	Length (mm)	Area (mm^2)
1.79	1.24	1.64	1.01	1.77	1.08
PK		PK		PK	
Head		Thorax		Legs	
Length (mm)	Area (mm^2)	Length (mm)	Area (mm^2)	Length (mm)	Area (mm^2)
1.74	1.23	1.75	1.09	1.78	1.17
WM		WM		WM	
Head		Thorax		Legs	
Length (mm)	Area (mm^2)	Length (mm)	Area (mm^2)	Length (mm)	Area (mm^2)
1.78	1.31	1.70	1.06	1.75	1.22
SWRQ		SWRQ		SWRQ	
Head		Thorax		Legs	
Length (mm)	Area (mm^2)	Length (mm)	Area (mm^2)	Length (mm)	Area (mm^2)
1.65	1.05	1.63	0.93	1.75	1.19
PQ		PQ		PQ	
Head		Thorax		Legs	

Length (mm)	Area (mm^2)	Length (mm)	Area (mm^2)	Length (mm)	Area (mm^2)
1.87	1.41	1.86	1.48	1.88	1.36

WF		WF		WF	
Head		Thorax		Legs	
Length (mm)	Area (mm^2)	Length (mm)	Area (mm^2)	Length (mm)	Area (mm^2)
1.77	1.16	1.72	0.99	1.68	0.99
